# Intraoperative measurement of intraventricular pressure in dogs with communicating internal hydrocephalus

**DOI:** 10.1371/journal.pone.0222725

**Published:** 2019-09-27

**Authors:** Malgorzata Kolecka, Daniela Farke, Klaus Failling, Martin Kramer, Martin J. Schmidt

**Affiliations:** 1 Department of Veterinary Clinical Sciences, Small Animal Clinic – Neurosurgery, Neuroradiology and Clinical Neurology, Justus-Liebig-University, Giessen, Germany; 2 Unit for Biomathematics and Data Processing, Faculty of Veterinary Medicine, Justus Liebig-University-Giessen, Giessen, Germany; Goethe University Hospital Frankfurt, GERMANY

## Abstract

Collapse of the lateral cerebral ventricles after ventriculo-peritoneal drainage is a fatal complication in dogs with internal hydrocephalus. It occurs due to excessive outflow of cerebrospinal fluid into the peritoneal cavity (overshunting). In most shunt systems, one-way valves with different pressure settings regulate flow into the distal catheter to avoid overshunting. The rationale for the choice of an appropriate opening pressure is a setting at the upper limit of normal intracranial pressure in dogs. However, physiological intraventricular pressure in normal dogs vary between 5 and 12 mm Hg. Furthermore, we hypothesise that intraventricular pressure in hydrocephalic dogs might differ from pressure in normal dogs and we also consider that normotensive hydrocephalus exists in dogs, as in humans. In order to evaluate intraventricular pressure in hydrocephalic dogs, twenty-three client owned dogs with newly diagnosed communicating internal hydrocephalus were examined before implantation of a ventriculo-peritoneal shunt using a single use piezo-resistive strain-gauge sensor (MicroSensor ICP probe). Ventricular volume and brain volume were measured before surgery, based on magnetic resonance images. Total ventricular volume was calculated and expressed in relation to the total volume of the brain, including the cerebrum, cerebellum, and brainstem (ventricle-brain index). Multiple logistic regression analysis was performed to assess the influence of the covariates “age”, “gender”, “duration of clinical signs”, “body weight”, and “ventricle-brain index” on intraventricular pressure. The mean cerebrospinal fluid pressure in the hydrocephalic dogs was 8.8 mm Hg (standard deviation 4.22), ranging from 3–18 mm Hg. The covariates “age”, (P = 0.782), “gender” (P = 0.162), “body weight”, (P = 0.065), or ventricle-brain index (P = 0.27)” were not correlated with intraventricular pressure. The duration of clinical signs before surgery, however, was correlated with intraventricular pressure (P< 0.0001). Dogs with internal hydrocephalus do not necessarily have increased intraventricular pressure. Normotensive communicating hydrocephalus exists in dogs.

## Introduction

Internal hydrocephalus is a common malformation of the central nervous system in dogs [1–3], characterised by the accumulation of cerebrospinal fluid (CSF) in the cerebral ventricles. This causes enlargement of the ventricular system at the expense of cerebral white matter and, later, subcortical and cortical grey matter [4–7]. Obstruction within the ventricular system or at the outflow through the lateral apertures of the fourth ventricle can stop CSF flow between the ventricular system and subarachnoid space, leading to a non-communicating form of hydrocephalus. Communicating hydrocephalus arises when impaired circulation, impaired absorption of CSF in the subarachnoid space, or both, occurs [2]. Other terms used in the classification of hydrocephalus in humans are “hypertensive” and “normotensive” to classify cases of hydrocephalus in which intraventricular pressure (IVP) is increased or normal.

Medical management of clinical signs involves reducing CSF production using carbo-anhydrase inhibitors, proton-pump inhibitors, or loop-diuretics, but these provide only temporary [8, 9], or no palliation of clinical signs [10]. Ventriculo-peritoneal shunting (VPS) has the potential for improvement of clinical signs and long-term survival in dogs and cats [8, 9, 11]. The shunt creates an alternative route for removal of CSF and restores the physiological balance between CSF production and absorption [2, 3]. However, VPS is fraught with high failure rates due to obstruction and insufficient drainage, infection of the ventricular catheter [8,9,11,12,13] and overdrainage, with subsequent subarachnoid haemorrhage and collapse of the cerebral hemispheres in both humans [14–16] and dogs [11–13, 17–19]. In order to avoid overshunting, the shunt systems contain valves that work depending on the differential pressure across them, which is termed the opening pressure. They act like on-off switches, opening when the IVP exceeds the valve’s opening pressure, allowing egress of CSF until IVP falls below the opening pressure. There are low- (0–5 cm of H_2_O), medium- (5–10 cm of H_2_O) and high-pressure valves (10–15 cm of H_2_O) [20]. Unfortunately, there are no evidence-based indications for the selection of appropriate valves in hydrocephalic dogs. The optimal valve pressure for the use in children and adults with hydrocephalus is usually the highest setting that still allows for the patient`s clinical improvement. If the preselected opening pressure does not adequately improve the clinical symptoms, modern systems allow the opening pressure to be gradually altered externally using a special magnetic adjusting device [21]. These shunt systems and the necessary equipment are extremely cost intensive, which limits their use in companion animals. Our rationale for the choice of an appropriate opening pressure is a setting at the upper limit of normal intracranial pressure (ICP) in dogs. We therefore use valves with an opening pressure of 15 cm H_2_0 (approximately 11 mm Hg) that allows egress of CSF at mildly increased IVP. The reduction of ventricular volume and associated improvement of clinical signs in a large cohort of operated dogs was somewhat variable [18]. This raises the question whether the selected opening pressure may not adequately address the requirements in each individual animal, and a valve with a lower pressure may be needed to reduce ventricular volume. Although IVP is one important determinant for the selection of the opening pressure of the valve, IVP in dogs with internal hydrocephalus has not been systematically investigated. The aim of this prospective cohort study was, therefore, to measure the IVP in dogs with primary communicating internal hydrocephalus. We hypothesise that IVP varies in hydrocephalic dogs, and that IVP is not necessarily increased in all dogs with hydrocephalus.

## Materials and methods

### Case selection and study criteria

Case selection criteria were the same as for previous studies [18] Client-owned dogs that presented between October 2017 and May 2019 in the Clinic for Small Animals at the Department for Veterinary Clinical Sciences, Justus-Liebig-University, Giessen, Germany, with newly diagnosed, communicating internal hydrocephalus were prospectively enrolled in the study. A detailed history was obtained for each dog to assess the duration of clinical signs. Clinical work-up included a standardised neurological examination performed by a board-certified neurologist (MK). Age, breed, gender, body weight and duration of clinical signs of each individual dog, as well as clinical signs, were recorded. To be included in the study, internal hydrocephalus had to be diagnosed as primary, based on the absence of other findings (e.g. malformations, inflammatory diseases) in magnetic resonance imaging, and communicating, based on the absence of a visible obstruction to CSF pathways.

### Magnetic resonance imaging (MRI)

MRI examinations were performed using a 3.0-T superconductive system (Siemens Verio) and sensitivity-encoding coil. A standardized MRI protocol was used as described in detail previously [18]. Sagittal, dorsal and transverse T2-weighted (TE = 120 ms, TR = 2900 ms), transverse FLAIR sequences (TE = 120ms, TI = 2400ms, TR = 7000ms) and transverse T1-weighted sequences (TR = 491 ms, TE = 8 ms) before and after contrast were acquired in all animals pre-operatively. Slice thickness varied from 2–3 mm depending on the size of the dog. Dogs were examined in dorsal recumbency. The field of view measured 180 x 180 mm in small dogs and 210 x 210 mm in large dogs. The matrix was 288 x 288 in small dogs and 384 x 384 in large dogs leading to a pixel size between 0.625 × 0.625 mm and 0.54 × 0.54 mm. Voxel size was not isotropic. MRI data sets of all dogs were evaluated as DICOM formatted images by use of an image viewer and processing software (EasyImage, easyVET, IFS GmbH, Hannover, Germany). Internal hydrocephalus was confirmed by the presence of distended lateral and/or third and fourth cerebral ventricles. In addition, thinning of the periventricular white matter, dorsal deviation of the corpus callosum, dilation of the olfactory bulb recesses, compression of the thalamic intermediate mass in the third ventricle and effacement of cerebral sulci were indicative for an active distension of the ventricles [22].

### Anaesthesia

A standard intravenous catheter (20 gauge) was placed in the right or left cephalic vein. Diazepam (0.5 mg/kg) was administered intravenously (IV), and anaesthesia was induced with propofol (2–4 mg/kg, IV). The anaesthetic protocol for MRI examination and surgical procedure included preoperative and postoperative opiate analgesia (levomethadon 0.5 mg/kg). Dogs were endotracheally intubated and anaesthesia was maintained with 1.5–2% isoflurane in oxygen. CO_2_ was measured using side stream capnography from the endotracheal tube. Animals were ventilated to maintain PaCO_2_ between 3–3.5%. During anaesthesia, systolic arterial blood pressure was measured with an oscillometric blood pressure monitor (Pulse Ox/NIBP 6004-SurgiVet). Measurements were obtained every 10 minutes. Systolic blood pressure less than 90 mm Hg was considered to indicate hypotension. Heart rate and body temperature were monitored continuously.

### IVP measurement

**Fig 1:**
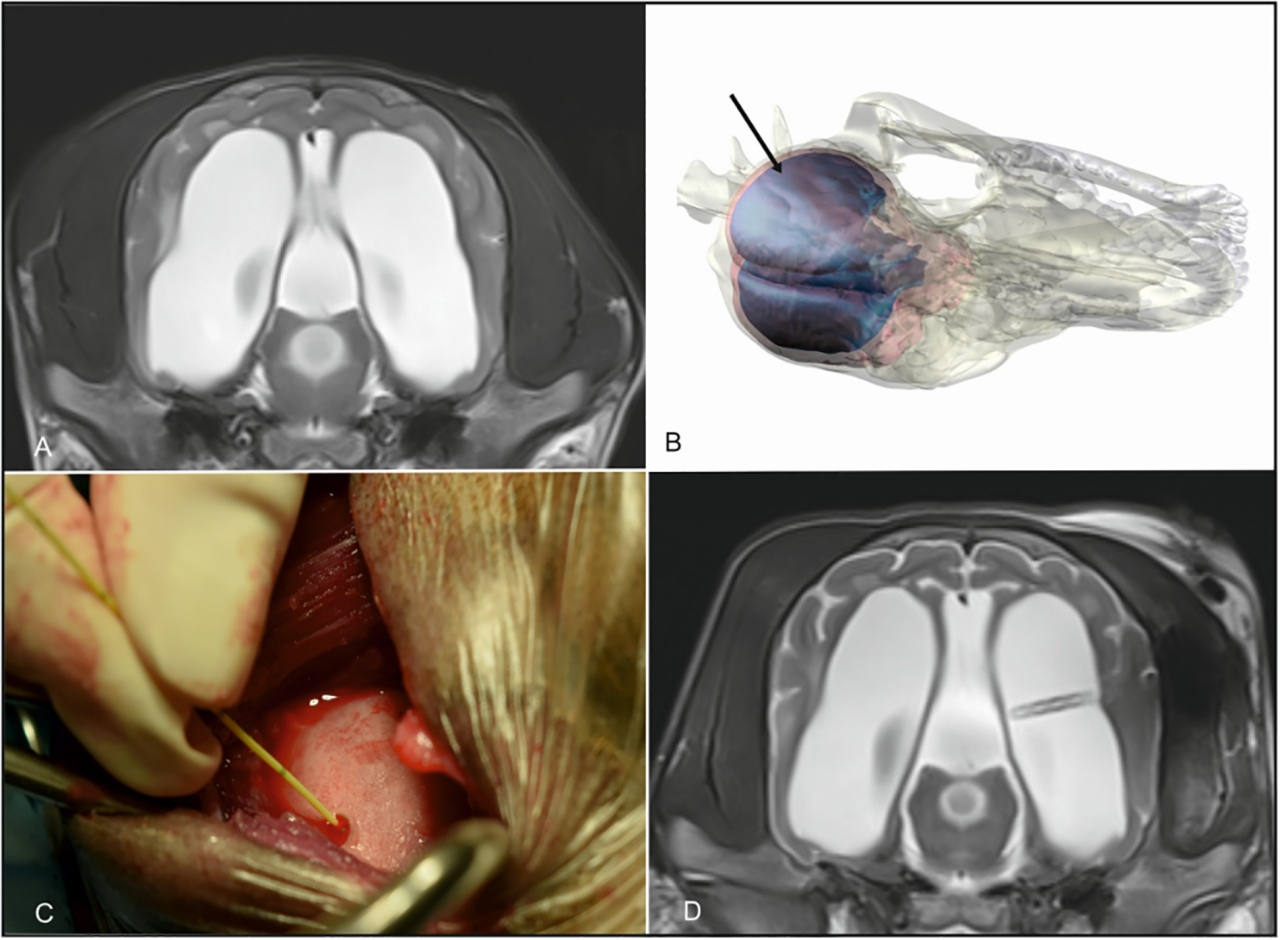
IVP measurement and Ventriculo-peritoneal shunt implantation and postoperative control in a Pomeranian with internal hydrocephalus. Maximum extension of the ventricles was determined in preoperative MRI (A). A small trephination was performed at the level of the caudal ectosylvian sulcus in the temporal lobe and the cortex was exposed after dural incision (B, black arrow) The sensor is advanced into the ventricle and IVP is measured (C). The same opening is used to implant the ventricular catheter. Proper implantation as controlled in postoperative MRI (D).

IVP was measured using a commercially available system for humans using a single use piezo-resistive strain-gauge sensor mounted in a miniature titanium case at the tip of a flexible nylon catheter (MicroSensor ICP probe). Pressure at the tip of the device results in an electrical voltage. Each dog was measured with a new individual pressure probe. Animals were placed in right lateral recumbency. A small trephination (7 mm) was performed at the level of the caudal ectosylvian sulcus in the temporal lobe (Fig 1A and 1B), after which the dura and cortex were coagulated using bipolar cautery. The sensor was zeroed against atmospheric pressure as specified by the manufacturer. Sensors were placed in 10 mL of sterile normal saline at a depth of 1 cm until a pressure of 0 mm Hg was observed. After calibration the sensor was placed into the ventricles (Fig 1C). Black lines on the transducer cable allow for assessment of distance. The cable was advanced to the level of the ventricles dependent on the size of the dog (1.5–2.5 cm). ICP readings were displayed using the Codman ICP EXPRESS monitor (Johnson&Johnson Medical GmbH; Codman, Norderstedt, Germany). Pressure was averaged over two minutes. The same opening was used to insert the ventricular catheter of the shunt system. Proper implantation of the ventricular catheter was controlled in postoperative MRI (Fig 1D).

### Morphometric procedures

**Fig 2:**
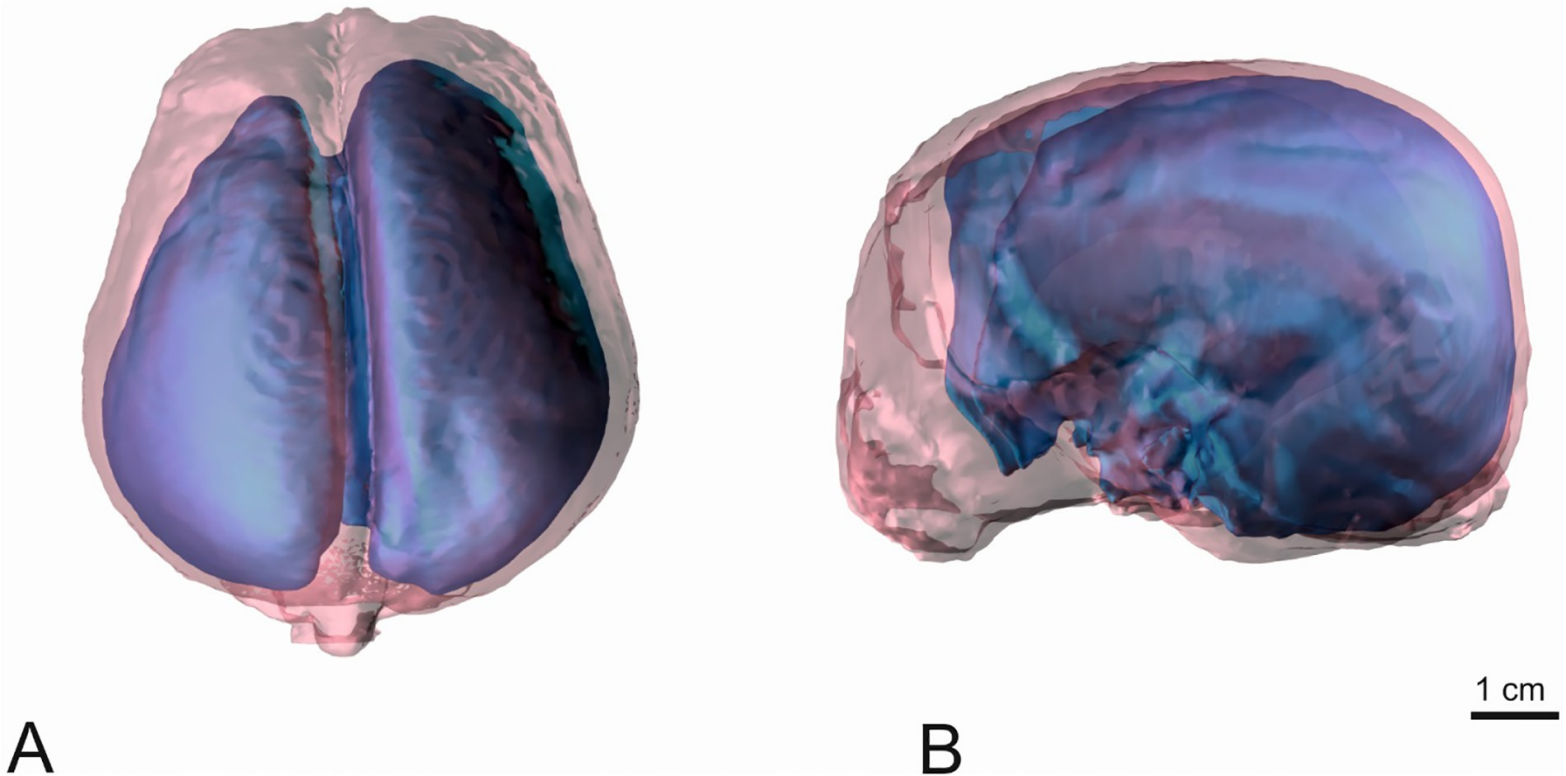
3D model of thebrain and ventricular volume of a Pomeranian with triventricular internal hydrocephalus. The voxels of the tissue of interest (skull: white, transparent, brain: red, transparent, lateral ventricles: blue) of each slice have been assembled and are displayed as a 3D model. A: dorsal view, B left lateral view.

In order to evaluate a possible relationship between ventricular size and IVP, total ventricular volume and brain volume was calculated from MR-images and expressed as the ventricle-brain index (V/B-index). Image processing for volume rendering was achieved using specialised graphical software, as described previously [18, 23] (AMIRA^®^, Mercury Computer Systems, Berlin, Germany). This program allows accurate manual image segmentation on a slice-by-slice basis. All voxels corresponding to a single anatomical structure (ventricular volume and brain) in the images were selected manually and assigned to the same value in the mask. The final mask thus contains information about all the selected anatomical structures in combination with the original data. The total ventricular volume comprised the ventricles, including the choroid plexus. The delineation of both the ventricular system and the brain parenchyma to the spinal cord was set along a vertical line at the obex. The segmented brain and ventricles are written and graphically presented by the programme (Fig 2) [23].

### Statistical analysis

Statistical analysis was performed using a commercial statistical software package (BMDP Statistical Software, Inc., Los Angeles, USA). Normality of the data was tested using the Shapiro-Wilk test. Because the statistical distribution of age and body weight was skewed to the right and the relationship to IVP was not linear the values were logarithmically transformed. The influence of the covariates “age”, “breed”, “gender”, “duration of clinical signs” and “V/B-index” were assessed using multiple logistic regression analysis. In a second step, the same potential associations were evaluated for each specific clinical sign using asymptotic linear regression analysis. As half of the dogs were Chihuahuas, IVPs of this breed were compared to the rest of the cohort using a t-test. The same was done to compare the IVPs of brachycephalic and mesocephalic dogs. Normality was tested using the Shapiro-Wilk test. A significance level of P< 0.05 was used.

### Ethical clearance

The study was conducted according to the University’s institutional animal welfare guidelines and in conformation with the German Animal Welfare Act. The study received ethical approval from the institutional ethics commissioner (Animal Welfare Officer, Justus-Liebig-University Giessen) and from the Official Veterinarian of the District Government of Giessen, reference number: V 54–19 c 20 15 h 02 GI 18/17 kTV 16/2018). Written consent was taken from the owners to include their animals into the study.

## Results

### Animals

Twenty-three dogs were included in the study. The majority of the dogs were Chihuahuas (n = 12). Six mesocephalic dogs and 17 brachycephalic dogs were examined. Eleven dogs were female, 12 dogs were male. Median age was 48 weeks (range 12–164). Breed, sex, age, body weight, duration of clinical signs, V/B-index and IVP of each individual dog are summarised in Table 1.

**Table 1:** Epidemiological data and results of volume- and intraventricular pressure measurements of dogs with internal hydrocephalus.

Dog Nr.	Breed	Sex	Age (weeks)	body weight (kg)	duration of signs (weeks)	V/B index	Intraventricular pressure (IVP)
**1**	**Chihuahua**	f	160	2	8	0.22	8 mm HG
**2**	**Chihuahua**	m	50	1.8	3	0.2	12 mm HG
**3**	**French bulldog**	f	20	9	2	0.29	18 mm HG
**4**	**Chihuahua**	m	16	1.5	6	0.28	4 mm HG
**5**	**West Highland White terrier**	m	14	8	8	0.3	9 mm HG
**6**	**Rhodesian Ridgeback**	f	12	17	1	0.32	18 mm HG
**7**	**French bulldog**	m	156	12	8	0.3	9 mm HG
**8**	**French bulldog**	f	24	9	10	0.36	10 mm HG
**9**	**Boder collie**	m	26	18	12	0.3	5 mm HG
**10**	**Chihuahua**	f	51	2.1	10	0.25	8 mm HG
**11**	**Chihuahua**	m	43	2.6	6	0.26	12 mm HG
**12**	**Chihuahua**	f	40	2.3	3	0.25	13 mm HG
**13**	**Cavalier King Charles spaniel**	f	164	12	2	0.31	14 mm HG
**14**	**Chihuahua**	f	20	2.5	12	0.25	9 mm HG
**15**	**Chihuahua**	m	22	2	12	0.22	5 mm HG
**16**	**Mini bullterrier**	m	48	18	1	0.33	18 mm HG
**17**	**Chihuahua**	f	143	2.3	10	0.32	8 mm HG
**18**	**Pomeranian**	f	48	9	12	0.36	5 mm HG
**19**	**Chihuahua**	f	50	2.5	12	0.25	4 mm HG
**20**	**Yorkshire terrier**	m	162	1.8	8	0.28	11 mm HG
**21**	**Maltese**	m	48	4	6	0.32	7 mm HG
**22**	**Chihuahua**	m	124	2.1	16	0.2	4 mm HG
**23**	**Chihuahua**	m	90	2.5	16	0.25	3 mm HG

### Intraventricular pressure

The mean IVP was 8.8 mm Hg (±4.22 mmHG), ranging from 3–18 mm Hg. Mean IVP of the Chihuahuas was 7.5 mm HG (± 3.5 mm HG), mean of the other breeds was 11.2 mm HG (± 5.02 mm HG). There was no statistical difference between the groups (P = 0.145). Mean IVP of the brachycephalic dogs was 8.9 mm HG (± 4.14 mm HG) and 10.33 (± 6.12 mm HG) in the mesocephalic dogs. Mean IVPs were not statistical different between the groups (P = 0.53). There was no influence of the variables age (P = 0.782), body weight (P = 0.065), gender (P = 0.162) or V/B index (P = 0.27) on IVP. The duration of signs on the other hand, negatively correlated with IVP (P< 0.0001), indicating a lower IVP with a longer duration of clinical signs.

## Discussion

Ventriculo-peritoneal shunt placement is the treatment of choice for the management of hydrocephalus in dogs [3, 8, 10, 11]. However, more than 50% of shunts in dogs fail and repeated neurosurgical operations are required [8, 11, 12]. Excessive shunt drainage is one serious complication of VPS that generates intracranial hypotension and ventricular collapse, in both humans [24, 25, 26], and dogs [8, 11]. Valves included into the shunt system regulate the flow through the shunt, and thereby avoid overshunting. Intraventricular CSF pressure must overcome the opening pressure of the valve to allow CSF drainage into the peritoneal cavity, which is why the IVP is one important information for the selection of an appropriate opening pressure. However, IVP in dogs with communicating hydrocephalus have never been investigated in a clinical setting until now.

In the present cohort of dogs with communicating hydrocephalus, the animals underwent IVP measurement directly before implantation of a VPS. A range of IVPs between 3 and 18 mm Hg were measured. Interestingly, only four dogs (18%) with internal hydrocephalus had pressure levels above the physiological upper range of IVP [27–31]. This raises the question whether intraventricular hypertension is at all necessary to maintain, or even to develop, ventricular distension in dogs. Modern theories for the pathogenesis of ventricular enlargement with patent connection between ventricles and the subarachnoid space suggest a reduced intracranial compliance as an underlying cause [32]. Following systolic expansion of the intracranial arteries, the following pressure wave is normally balanced by expulsion of venous blood, as well as CSF, through the foramen magnum [33]. The cranial capacity, i.e., the part of the cranial cavity that is not occupied by the brain, is reduced in brachycephalic dogs [34] that are mostly affected by internal hydrocephalus [1]. Obstruction of the basal CSF cisterns in these breeds might impair dissipation of systolic pulsation waves of the basal arteries into the subarachnoid space [33]. The consequence can be a stronger arterial pulsation in the choroidal arteries and the choroid plexus and hyperdynamic CSF pressure waves that can cause ventricular expansion independent of increased pressure [35–37]. Communicating hydrocephalus could be created in animal models by merely increasing the amplitude of the intraventricular CSF pulsations, leaving the mean IVP unchanged [37–39] but creating a transient minimal pressure gradient between the ventricles and the subarachnoid space (transmantle gradient) of approximately 0.5 mm HG [40–43].

The finding of normal or low IVP in the majority of the dogs in this study could also be dependent on the time point of measurement. It is also possible that an initially high IVP returns to normal values in the late course of the disease. In experimental hydrocephalus in dog models, an initial rise up to 60 mm Hg subsided to normal IVP values within a week [40, 44]. The authors state that IVP begins to fall due to increasing parenchymal compliance producing sustained ventriculomegaly at normal IVP. The documented correlation between a longer duration of clinical signs and lower IVP in this study might support the hypothesis that IVP returns to normal levels, if the parenchyma yields under the pressure. Ventricular enlargement with IVP below normal values (low-pressure hydrocephalus) was suggested to be related to an additional alteration of the viscoelastic properties of the brain at a later stage of ventricular dilation. Ongoing ventricular distension alters the flow and content of parenchymal water [45]. With increasing IVP and disruption of the ependymal lining, the physiological flow of extracellular fluid from the parenchyma towards the ventricle reverses, and brain water content increases [46]. This state is associated with decreased brain elasticity [47]. With time, interstitial fluid is driven into brain capillaries and absorbed to the circulation, which increases brain compliance, thereby lowering IVP despite ventricular distension [48].

ICP monitoring techniques are multiple and diverse [49]. Measurements can be performed in the intraventricular, intraparenchymal, epidural, or subdural compartment. Intraparenchymal or intraventricular monitoring is the standard choice in the clinical setting [50]. Intraparenchymal pressure monitoring provides equivalent measurements when compared to intraventricular assessment, which is in fact the gold-standard for ICP monitoring in humans [51–54]. Consistent values have also been measured in anaesthetised, clinically sound, dogs using ventricular ICP measurements [55] as compared to intraparenchymal-, [28, 56], or cisterna magna measurements [31, 57]. The Codman MicroSensor has been shown to provide reliable and accurate ICP measurements in dogs [28]. We have measured IVP under general anaesthesia. Most inhalant anaesthetics cause a minimal-to-moderate increase in ICP [58–60], which may bias its measurement during VPS surgery. However, non‐anaesthetised dogs had mean ICPs in a range consistent with values of previous reports in dogs under general anaesthesia [28], suggesting a minimal effect of these agents. Alteration of cerebral perfusion pressure, which in turn is dependent on mean arterial blood pressure, has a major effect on ICP. As blood pressure and CO_2_ both have a major influence on IVP, these parameters were held constant during surgery.

**Fig 3:**
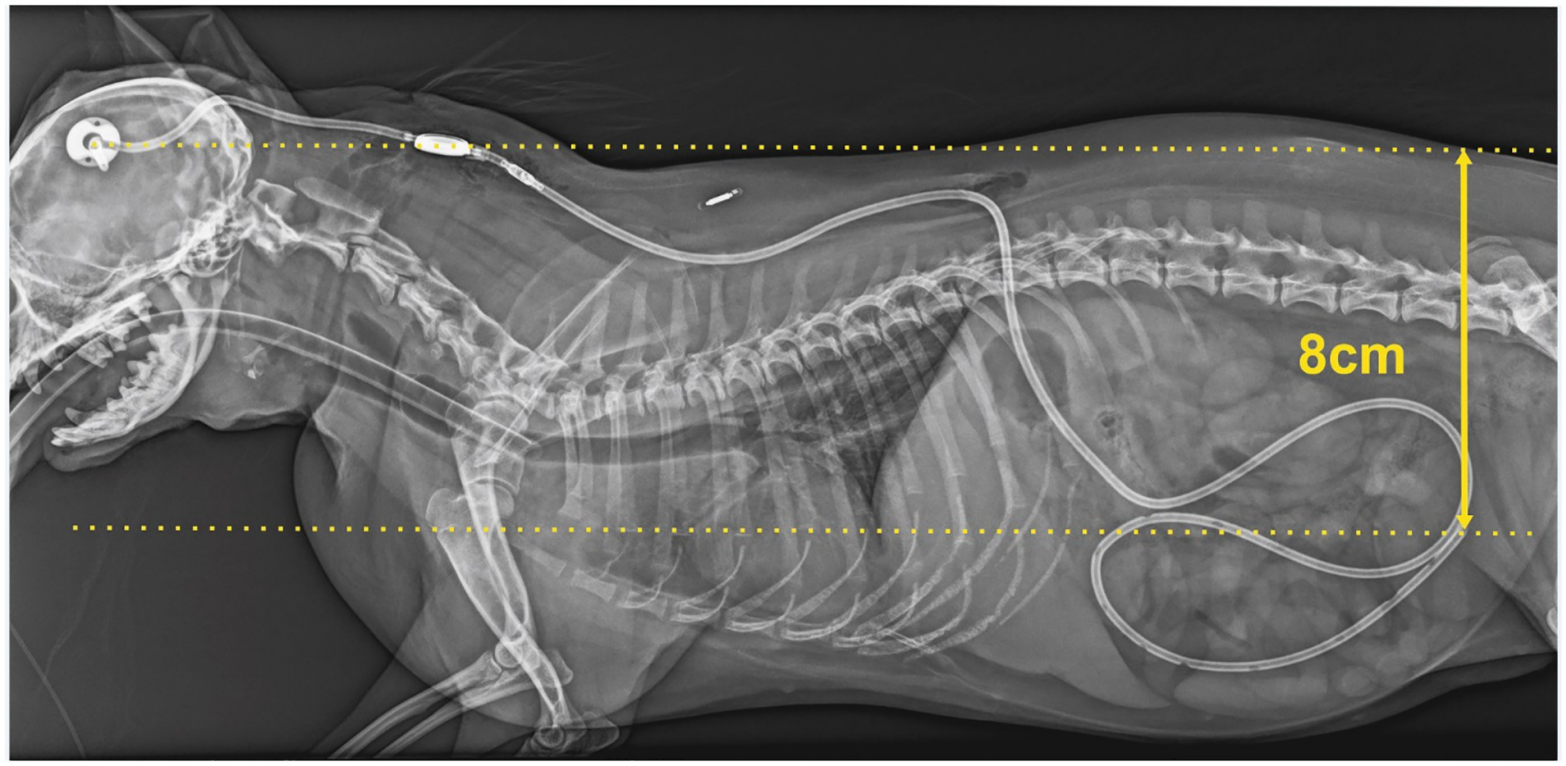
Postoperative latero-lateral radiograph after Ventriculo-peritoneal shunt implantantion of a Chihuahua (dog No. 11; Table 1). The dotted lines illustrate the distance between the ventricular catheter and tip of the peritoneal catheter.

IVP is a dynamic parameter and measurement at one time-point only represents a snapshot. ICP measurements in awake dogs were found to differ depending on head position [28]. Mean values for ICP increased by >100% comparing head elevation to head down positions [28]. These effects, and the influence of other normal activities (e.g., physical exercise, coughing and sneezing, defecation, etc.), on IVP of hydrocephalic dogs have not been established. The possible change of IVP and other pressures with influence on the opening pressure of the valve has important implications for VPS treatment. The drainage rate of the shunt system is not only determined by the IVP, but also by the intraabdominal pressure, and the hydrostatic pressure column in the catheter [61]. Drainage of fluid from the ventricles into the peritoneal cavity is subject to gravity acting upon flow, according to the body position. In humans, over-drainage mostly occurs in a standing position, when the hydrostatic pressure in the vertical catheter causes a siphon effect [62]. In dogs, most of the shunt system runs along the horizontal body axis, but the effect of gravitational forces can certainly not be ignored. The difference between the tip of the intraventricular and intraperitoneal end varies between 5–40 cm depending on the size of the dog (Fig 3). The intraabdominal pressure varies between 0–7 mm Hg in dogs with a mean of 4.5 cm H_2_0 (approximately 3.2 mm Hg) [63, 64]. If the opening pressure of a valve is dependent on IVP, hydrostatic pressure and intraabdominal pressure, which are all variable and dynamic in the awake dog, it is logical to expect a constant unpredictable change of flow through the shunt system, with possible fatal complications. Furthermore, if the brain parenchyma re-expands after a while and brain compliance decreases IVP might change again as an effect of shunting.

The results of this study highlight the need for a better understanding of how ventricular drainage is influenced in hydrocephalic dogs. In order to avoid complications of over-, and under-shunting and reduce the morbidity and mortality of dogs with hydrocephalus after shunting, IVP should be constantly monitored in the awake dog and adapted based on the actual conditions, if necessary.

## Conclusion

Dogs with internal communicating hydrocephalus can have variable IVPs. It can be elevated, within normal ranges, and even below normal physiological values.

## Abbreviations

CSF: cerebrospinal fluid
FLAIR: fluid attenuated inversion recovery
ICP: intracranial pressure
IV: intravenously
IVP: intraventricular pressure
MRI: Magnetic resonance imaging
V/B index: Ventricle-brain index
VPS: ventriculo-peritoneal shunting
